# Amino Acid Metabolism of *Thermoanaerobacter* Strain AK90: The Role of Electron-Scavenging Systems in End Product Formation

**DOI:** 10.1155/2015/410492

**Published:** 2015-08-27

**Authors:** Sean Michael Scully, Johann Orlygsson

**Affiliations:** Faculty of Natural Resource Sciences, University of Akureyri, Borgir, Nordurslod 2, 600 Akureyri, Iceland

## Abstract

The catabolism of the 20 amino acids by *Thermoanaerobacter* strain AK90 (KR007667) was investigated under three different conditions: as single amino acids without an electron-scavenging system, in the presence of thiosulfate, and in coculture with a hydrogenotrophic methanogen. The strain degraded only serine without an alternative electron acceptor but degraded 11 amino acids (alanine, cysteine, isoleucine, leucine, lysine, methionine, phenylalanine, serine, threonine, tyrosine, and valine) under both of the electron-scavenging systems investigated. Acetate was the dominant end product from alanine, cysteine, lysine, serine, and threonine under electron-scavenging conditions. The branched-chain amino acids, isoleucine, leucine, and valine, were degraded to their corresponding fatty acids under methanogenic conditions and to a mixture of their corresponding fatty acids and alcohols in the presence of thiosulfate. The partial pressure of hydrogen seems to be of importance for the branched-chain alcohol formation. This was suggested by low but detectable hydrogen concentrations at the end of cultivation on the branched-chain amino acid in the presence of thiosulfate but not when cocultured with the methanogen. A more detailed examination of the role of thiosulfate as an electron acceptor was performed with *Thermoanaerobacter ethanolicus* (DSM 2246) and *Thermoanaerobacter brockii* (DSM 1457).

## 1. Introduction

*Thermoanaerobacter* and* Caldanaerobacter* species have been intensively investigated in the context of biofuel production due to their broad substrate spectrum, especially among the sugars present in lignocellulosic biomass, and due to their high ethanol and hydrogen yields [1–4]. However, the metabolism of both proteins and amino acids by thermophilic bacteria has received much less attention.

Branched-chain amino acids (BCAAs) are known to be degraded to their corresponding branched-chain fatty acids (BCFAs) under anaerobic conditions [5, 6]. The majority of catabolic studies of BCAAs have focused on aerobic bacteria such as species of* Staphylococcus* and* Enterococcus* [7–9], aerotolerant anaerobes including* Lactobacillus sakei* [10], or yeasts that use the so-called Ehrlich pathway [11, 12]. These studies have often focused on the formation of compounds that contribute to the flavor profile of foods and beverages (branched-chain and aromatic aldehydes, alcohols, and acids) [13, 14]. Amino acid metabolism has been investigated in some detail for* Thermoanaerobacter brockii* which degrades the BCAAs (isoleucine, leucine, and valine) by using an oxidative deamination and decarboxylation mechanism, but only in the presence of a hydrogen-scavenging system [6]. An external electron acceptor is required due to the unfavorable thermodynamics; Δ*G*°' for the degradation of these three BCAAs is between +4.2 and +9.7 kJ/mol [6, 15]. However, the addition of thiosulfate or coculturing amino acid degrading thermoanaerobes with hydrogen-scavenging methanogens allows for the degradation of these amino acids to their corresponding fatty acids [6]. Thus, leucine is degraded to 3-methylbutyrate, isoleucine to 2-methylbutyrate, and valine to 2-methylpropionate. Serine, however, is degraded to acetate, ethanol, hydrogen, and carbon dioxide by* T. brockii* without an external electron acceptor. The addition of an external electron acceptor, however, shifts the fermentation products of this amino acid to acetate and greatly reduces ethanol formation [6]. Recent investigation in our research group showed that most species within the genera of* Thermoanaerobacter* and* Caldanaerobacter* degrade the BCAAs not only to their corresponding fatty acids but also to a mixture of BCFAs and branched-chain alcohols (BCOHs) under thiosulfate reducing conditions [16, 17].

The present study focuses on the amino acid catabolism of* Thermoanaerobacter* AK90, which was isolated from a hot spring in Iceland. Special emphasis was given to amino acid degradation by the strain in the presence of thiosulfate or in coculture with a hydrogenotrophic methanogen,* Methanothermobacter* M39. For comparison, the degradation of BCAAs by* Thermoanaerobacter ethanolicus* (DSM 2246) and* Thermoanaerobacter brockii* (DSM 1457) was investigated under the same growth conditions as well as using different concentrations of thiosulfate to reveal its effect on end product distribution between the corresponding BCFA and BCOH.

## 2. Materials and Methods

### 2.1. Bacterial Strains

*Thermoanaerobacter* AK90 (KR007667) was isolated from a hot spring in Grensdalur (Southwest Iceland) using Timothy grass (*Phleum pratense*) hydrolysate as a carbon source according to methods already described [18].* Thermoanaerobacter ethanolicus* (DSM 2246) and* Thermoanaerobacter brockii* (DSM 1457) were purchased from Deutsche Sammlung von Mikroorganismen und Zellkulturen (DSMZ). The hydrogenotrophic methanogen,* Methanothermobacter* strain M39, used in coculture experiments was isolated as described earlier [19].

### 2.2. Phylogenic Characterization

Phylogenetic characterization of strain AK90 has been described earlier [17]. The* Methanothermobacter* strain M39 was analyzed for 16S rDNA by DSMZ. Genomic DNA extraction was carried out using MasterPure Gram Positive DNA Purification Kits from Epicentre Biotechnologies, Germany, according to the manufacturer's instructions. PCR mediated amplification of the 16S rDNA and purification of the PCR product was carried out as previously described [20]. Purified PCR products were sequenced using the BigDye Terminator v1.1 Cycle Sequencing Kit (Applied Biosystems) as described in the manufacturer's protocol. Sequence reactions were electrophoresed using the 3500 xL Genetic Analyzer from Applied Biosystems. The resulting sequence data was put into the alignment editor ae2 [21], aligned manually, and compared with representative 16S rRNA sequences of organisms belonging to Archaea [21]. For comparison, 16S rRNA sequences were obtained from the EMBL database or RPD [21].

### 2.3. Culture Conditions

The medium (per liter), hereafter referred to as BM medium, consisted of NH_4_Cl 0.3 g, NaCl 0.3 g, CaCl_2_ 0.11 g, MgCl_2_ × 6H_2_O 0.1 g, yeast extract 2.0 g, resazurin 1 mg, trace element solution 1 mL, vitamin solution 1 mL, and NaHCO_3_ 0.8 g. Phosphate buffers were also used where 1 M stock solutions of NaH_2_PO_4_ and Na_2_HPO_4_ were made and added to the media to give a buffer capacity of 50 mM. The vitamin solution was prepared according to DSM141. The trace element solution consists of (g/L) FeCl_2_ × 4H_2_O, 2.0, EDTA, 0.5, CuCl_2_, 0.03, H_3_BO_4_, ZnCl_2_, MnCl_2_ × 4H_2_O, (NH_4_)_6_Mo_7_O_24_ × 4H_2_O, AlCl_3_, CoCl_2_ × 6H_2_O, NiCl_2_, all 0.05 mg, and 1 mL of concentrated HCl. The medium was prepared by adding the phosphate buffer, yeast extract, and resazurin to distilled water, which was then boiled for 5–10 min and cooled while flushing with nitrogen. The mixture was then transferred to cultivation bottles and autoclaved for 60 minutes. All other components of the medium were added separately through filter-sterilized solutions. The gas phase in all fermentation experiments consisted of 5.0 nitrogen (<5 ppm O_2_). All experiments were performed at 65°C and pH 7.0 without agitation. When* Methanothermobacter* M39 was used in a coculture with other strains, it was pregrown on hydrogen and carbon dioxide (80/20 v/v) for one week. Prior to inoculating strain AK90 into the methanogenic cultures, the bottles were flushed with nitrogen. The inoculum volume of strain AK90 was 2% (v/v) from the exponential growth phase of stock cultures grown on glucose (20 mM) in all cases. Substrate solutions were added to culture media after autoclaving (121°C for 60 minutes) through a syringe filter (Whatman PES, 0.45 *μ*m). All experiments were done in duplicate.

### 2.4. Degradation of Amino Acids

The ability of strain AK90 to utilize amino acids was tested using the BM medium supplemented with different amino acids (20 mM) in the presence or absence of thiosulfate (40 mM), or in a coculture with* Methanothermobacter* M39. The samples were grown for five days, at which time liquid (1 mL) and gas (0.2 mL) samples were withdrawn and the end products analyzed. Fermentation of single amino acids and amino acids in the presence of thiosulfate was performed in 24.5 mL serum bottles with a liquid-gas phase ratio of 1 : 1. Experiments with amino acid degradation in the presence of the methanogen were performed in 117.5 mL serum bottles with a liquid-gas ratio of 0.2. Similar procedure was used for investigating the growth of* T. brockii* and* T. ethanolicus* on BCAAs (20 mM) in the presence of the methanogen, but when thiosulfate was used, initial concentrations of thiosulfate varied between 5 and 80 mM.

### 2.5. Analytical Methods

Hydrogen and methane were analyzed using a PerkinElmer Auto System XL gas chromatograph equipped with a thermoconductivity (TCD) detector. Nitrogen was used as a carrier gas at a rate of 3 mL/min, with another 17 mL/min as make-up gas. The column used was Supelco 1010 Carboxen GC Plot Capillary Column. The oven temperature was 80°C, and the injector and detector temperatures were kept at 200°C. Alcohols and volatile fatty acids were measured by gas chromatography using a PerkinElmer Clarus 580 gas chromatograph equipped with a flame ionization detector (FID) using standards purchased from Sigma Aldrich. The column used was 30 m DB-FFAP capillary column (Agilent Industries Inc., Palo Alto, CA, USA). Amino acids were analyzed using the ninhydrin method by mixing 100 *μ*L of sample and 100 *μ*L of 1% (w/v) ninhydrin reagent (60% v/v 2-propanol and 40 mM acetate buffer, pH 5.5) in a microtiter plate and incubating at 100°C for 20 minutes. After cooling, 200 *μ*L of 50% (v/v) 2-propanol was added and the absorbance read at 580 nm on a Bioscreen C (Oy Growth Curves AB, Finland). Hydrogen sulfide was analyzed according to the method described by Cord-Ruwisch [22]. Thiosulfate was analyzed according to Westley [23] modified for use in microplates. Sulfur was detected by microscopic examination.

## 3. Results

### 3.1. Strains AK90 and M39

Strain AK90 was isolated from a hot spring in Iceland [17]. According to partial 16S rRNA sequence data, the strain belongs to the genus* Thermoanaerobacter* with the most closely related strains being* T. thermohydrosulfuricus* (100.0% similarity),* T. ethanolicus* (99.7%), and* T. pseudoethanolicus* (99.5%). Strain M39 was also isolated from a hot spring in Iceland [19]. According to partial 16S rRNA (about 800 nucleotides), the strain belongs to the genus* Methanothermobacter* and is most closely related to* M. marburgensis* Marburg^T^ (99.7% similarity).

### 3.2. Degradation of Amino Acids by Strain AK90 without an External Electron Acceptor

The following amino acids were not degraded under any culture conditions by strain AK90: arginine, asparagine, aspartic acid, glycine, glutamate, glutamine, histidine, proline, and tryptophan. Of the 20 amino acids tested as single substrates, only serine was degraded (Table 1). Serine (16.0 mM) was degraded to ethanol (5.7 mM), acetate (15.7 mM), hydrogen (9.7 mmol/L), and carbon dioxide (calculated; 21.4 mmol/L). When the quantity of end products in the control bottles (yeast extract) was subtracted from these values, a carbon balance of 87.5% was obtained. Other amino acids were only degraded to a small extent with end product formation similar to or slightly higher than that observed in the control bottles.

### 3.3. Degradation of Amino Acids in the Presence of* Methanothermobacter* M39

During degradation of amino acids in the presence of* Methanothermobacter* M39, strain AK90 was capable of degrading a much wider spectrum of amino acids as compared to cultures without any electron-scavenging system (Table 1). The strain converted more than 90% of cysteine, isoleucine, methionine, phenylalanine, serine, threonine, and tyrosine. Additionally, between 11.5 and 16.7 mM (57.5 to 83.5%) of alanine, leucine, lysine, and valine were degraded. End products from alanine, cysteine, lysine, serine, and threonine were primarily acetate, although small amounts of butyrate (2.3 mM) were produced from lysine.

The BCAAs were degraded to their corresponding BCFAs (leucine to 3-methylbutyrate, isoleucine to 2-methylbutyrate, and valine to 2-methylpropionate). Methane concentrations from BCAA fermentation were similar, ranging from 8.9 to 11.7 mmol L^−1^ (Table 1). The stoichiometry for the BCAAs under methanogenic conditions was as follows (control subtracted):

 1.00 leucine → 0.71 3-methylbutyrate + 0.38 CH_4_
 1.00 isoleucine → 0.77 2-methylbutyrate + 0.47 CH_4_
 1.00 valine → 0.72 2-methylpropionate + 0.60 CH_4_

Methane production by a hydrogenotrophic methanogen uses 4 moles of hydrogen to produce 1 mole of methane. The oxidative deamination and decarboxylation from one mole of a single BCAA should thus yield 0.5 moles of methane since, for each mole of degraded BCAA, 2 moles of hydrogen are produced [6]. This is reasonably consistent with the data obtained.

The aromatic amino acids phenylalanine and tyrosine, as well as methionine, were almost completely degraded under methanogenic conditions. Several unidentified peaks were observed late in the gas chromatograph run (between 10 and 14 min) from these amino acids and methane concentrations were similar as produced from the BCAAs. Ethanol was only a minor product from all amino acids as is shown in Table 1 and usually in similar concentrations as was observed in control bottles.

### 3.4. Degradation of Amino Acids in the Presence of Thiosulfate

The addition of thiosulfate in the present investigation resulted in similar degradation spectra as observed for methanogenic conditions. Six amino acids were completely degraded (alanine, cysteine, isoleucine, leucine, serine, and valine). Degradation of methionine, phenylalanine, and tyrosine was between 11.7 and 16.4 mM (58.7 to 82.0%), but lysine and threonine were only partially degraded (Table 1). As for methanogenic cultures on alanine, cysteine, and serine, acetate was the major end product under thiosulfate conditions. Similarly, unidentified peaks were observed from methionine, phenylalanine, and tyrosine. In all experimental bottles, the thiosulfate concentrations were negligible (<0.2 mM) at the end of cultivation and hydrogen sulfide and sulfur were end products from thiosulfate reduction, but only hydrogen sulfide was quantified. The concentrations of hydrogen sulfide from the BCAAs were between 12.4 and 13.5 mM.

During the degradation of the BCAAs under these conditions, not only the corresponding BCFAs but also the BCOHs were produced. Thus, leucine was degraded to a mixture of 3-methylbutyrate and 3-methylbutanol, isoleucine to 2-methylbutyrate and 2-methylbutanol, and valine to 2-methylpropionate and 2-methylpropanol. The stoichiometry for the BCAAs degradation was as follows (controls subtracted):

 1.00 leucine → 0.49 3-methylbutyrate + 0.18 3-methylbutanol + 0.47 H_2_S
 1.00 isoleucine → 0.78 + 2-methylbutyrate + 0.22 2-methylbutanol + 0.60 H_2_S
 1.00 valine → 0.95 2-methylpropionate + 0.09 2-methylpropanol + 0.60 H_2_S

In all cases, the branched-chain fatty acid concentration was greater than that of the corresponding alcohol.

### 3.5. Degradation of Branched-Chain Amino Acids by* Thermoanaerobacter ethanolicus* and* Thermoanaerobacter brockii*

The type species of the genus* Thermoanaerobacter* is* T. ethanolicus* ^T^ (DSM 2246) and was investigated in this study as well as* T. brockii* (DSM 1457) for the ability to produce BCOHs (and BCFAs) from BCAAs. These species were cultivated at 20 mM concentrations of the BCAAs both in the presence and absence of electron-scavenging systems as was done for strain AK90, but the initial concentration of thiosulfate varied between 5 and 80 mM. When cultivated without any electron-scavenging system, the BCAAs were only degraded to a minor extent, producing low amounts of BCFAs (<2.0 mM) (Figures 2 and 3). An earlier study performed in our laboratory on* T. brockii* showed that, under methanogenic conditions, the BCAAs (20 mM) were almost completely degraded and the production of BCFAs varied between 14.8 and 15.9 mM, and similar amounts of methane were produced as compared to strain AK90 (between 7.6 and 9.3 mmol/L) (Table 2) [18]. A similar pattern was also observed for* T. ethanolicus* when cocultivated with the methanogen on leucine and isoleucine; almost complete degradation of the BCAAs and the amounts of the BCFAs were 15.0 and 14.3 mM for leucine and isoleucine, respectively; methane was observed in similar concentrations (7.4 to 7.8 mmol/L) as before (Table 2). Valine, however, was only partially degraded under these conditions by* T. ethanolicus*, resulting in lower concentrations of BCFAs and methane yields (Figure 3 and Table 2).

Similarly, cultivation with thiosulfate as an electron scavenger by these two species resulted in the production of BCFAs from BCAAs (20 mM), as well as the formation of their corresponding BCOHs (Figures 2 and 3). However, the amount of alcohol formation was lower as compared to strain AK90. By increasing the concentration of thiosulfate from 5 to 80 mM, an increase in the BCFA formation as well as in amino acid degradation was observed for* T. brockii* (Figures 2(a)–2(c)). At lower concentrations of thiosulfate, the electron sink is not in excess, leading to hydrogen accumulation and inhibition of further amino acid degradation. The highest amounts of BCOHs were produced under these conditions. By increasing the initial thiosulfate concentration, hydrogen was kept at lower concentrations, allowing for almost complete degradation of the amino acids. The concentration of hydrogen sulfide is not correlated to increased initial thiosulfate concentrations except for the step from 5 to 10 mM thiosulfate concentrations. At higher thiosulfate concentrations, H_2_S actually decreased in the experimental bottles; microscopic observations as well as the formation of a strong yellow color, especially at high initial thiosulfate loadings, showed the presence of elemental sulfur.

*T. ethanolicus* produced much lower amounts of BCFAs and BCOHs compared to strains AK90 and* T. brockii*, in the presence of thiosulfate with only between 3.0 and 10.0 mM of the amino acids being degraded (Figures 3(a)–3(c)). Hydrogen concentrations at high initial thiosulfate concentrations were also higher as compared to* T. brockii*. Hydrogen sulfide and BCOHs concentrations showed similar spectrum as with* T. brockii*, increasing from 5 to 10 mM initial thiosulfate concentrations, but decreasing at higher concentrations. These values, however, were always found to be in lower concentrations as compared with* T. brockii*.

## 4. Discussion

The amino acid metabolism of thermophilic bacteria has been investigated previously [5, 6, 24, 25], though to a lesser extent as compared to the metabolism of carbohydrates. Many of these investigations focus on the thermodynamics of amino acid degradation. BCAAs can only be degraded when the electrons are scavenged either by the addition of thiosulfate or by coculturing with a methanogen [6] or with sulfate reducing bacteria [26]. Strain AK90 could only degrade one amino acid (serine) when cultivated without any electron-scavenging system; the end product under these culture conditions was predominantly acetate and 80% of the amino acid was degraded (Table 1). When serine was degraded in the presence of thiosulfate or in a coculture with* Methanothermobacter* M39, complete degradation occurred and increased acetate concentrations were observed. This is similar to a study in which* T. brockii* was grown on serine where a shift from ethanol to acetate was reported under hydrogen-scavenging conditions [6]. For most of the other amino acids, only a small fraction is degraded with the accumulation of hydrogen leading to thermodynamic hindrance for further degradation.

Under methanogenic conditions, much wider spectra of amino acids were degraded (Table 1). This is in agreement with previous studies by others where proteins are degraded to a greater extent under methanogenic conditions [15, 27]. This was most clearly shown during the degradation of the BCAAs where more than 80% of them were degraded to their corresponding BCFAs. As stated previously,* Thermoanaerobacter* strain AK90 threonine was not degraded as a single substrate but under methanogenic conditions it was almost completely converted to acetate (Table 1). This implies that its degradation pathway is more complex and it has been shown in other studies that threonine is degraded to propionate or to a mixture of propionate and butyrate [5, 28]. The amount of methane produced in experimental bottles supplemented with the aromatic amino acids and methionine was between 6.3 and 8.2 mmol/L which was lower as compared to methane from the BCAAs (between 8.9 and 11.1 mmol/L), but well above the control value (yeast extract only). This, together with the fact that these four amino acids were almost completely degraded under these conditions, indicates an oxidative mechanism for their initial degradation pathways. This was also indicated by a number of unidentified products observed during the gas chromatography run from these amino acids. Thus, methionine is likely to be degraded to 3-methylthiopropionate, phenylalanine to 2-phenylethanoate, and tyrosine to 2-(4-hydroxyphenyl)ethanoate. It is known that these end products have been produced by anaerobic bacteria [29].

Thiosulfate reduction to sulfide and sulfur is a common characteristic among the genera* Thermoanaerobacter*,* Caldanaerobacter*, and* Thermoanaerobacterium* [6, 24, 30]. Fardeau and coworkers demonstrated a shift in end product formation by* Thermoanaerobacter finnii* (now* T. brockii* subsp.* finnii*) on glucose in the presence and absence of thiosulfate [24]. Both ethanol and lactate decreased during thiosulfate reduction to hydrogen sulfide, while acetate and biomass increased. The influence of using hydrogen-scavenging systems has also been investigated during the amino acid degradation by* Thermoanaerobacter brockii* [6]. Both thiosulfate and the presence of a hydrogen-scavenging methanogen were crucial for the oxidative deamination of the BCAAs by this strain.

**Figure 1:**
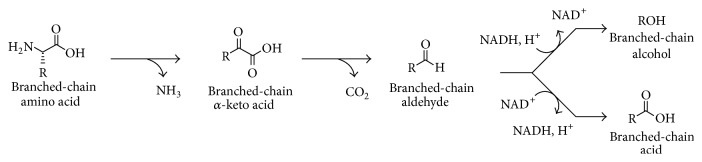
The Ehrlich pathway (from [17]). Catabolism of branched-chain amino acids (leucine, isoleucine, and valine) leading to the production of branched-chain acids and alcohols.

During the degradation of the BCAAs in the presence of thiosulfate, strain AK90 produced not only the corresponding fatty acids, but also their corresponding alcohols (Table 1). This has only recently been shown by some species within the genera of* Thermoanaerobacter* and* Caldanaerobacter* [16, 17] and is known to occur in lactic acid bacteria [10] and yeasts [12] through the Ehrlich pathway (Figure 1). The reason for a mixture of a fatty acid and alcohol produced from the BCAAs can most likely be directly linked to the partial pressure of hydrogen and regulation of the NADH/NAD^+^ and the corresponding hydrogenases are involved. Although the concentrations of hydrogen are very low under both electron-scavenging systems (methanogenic/thiosulfate), it was detectable under thiosulfate reduction conditions which may explain the formation of the reduced alcohol.

Interestingly, threonine was only partially (5.5 mM) degraded under methanogenic conditions by strain AK90 (Table 1). This is in contrast to the relatively large amounts of acetate produced. The reason might be that the strain is using a pathway postulated by Barker [31]: formation of acetate from threonine can be accomplished by direct cleavage of threonine to acetaldehyde and glycine by threonine aldolase reaction, followed by oxidation of acetaldehyde to acetate and the conversion of glycine to acetate. Since glycine is not utilized as a single substrate under any culture conditions by strain AK90, it could be accumulating in the culture broth (assuming this pathway is active), thus explaining the seemingly partial degradation for threonine. From the H_2_S and S formation observed during growth on phenylalanine, tyrosine, and methionine in the presence of thiosulfate and the fact that the same unidentified peaks were also observed as under methanogenic conditions, it can be deduced that similar end products were produced.

**Figure 2:**
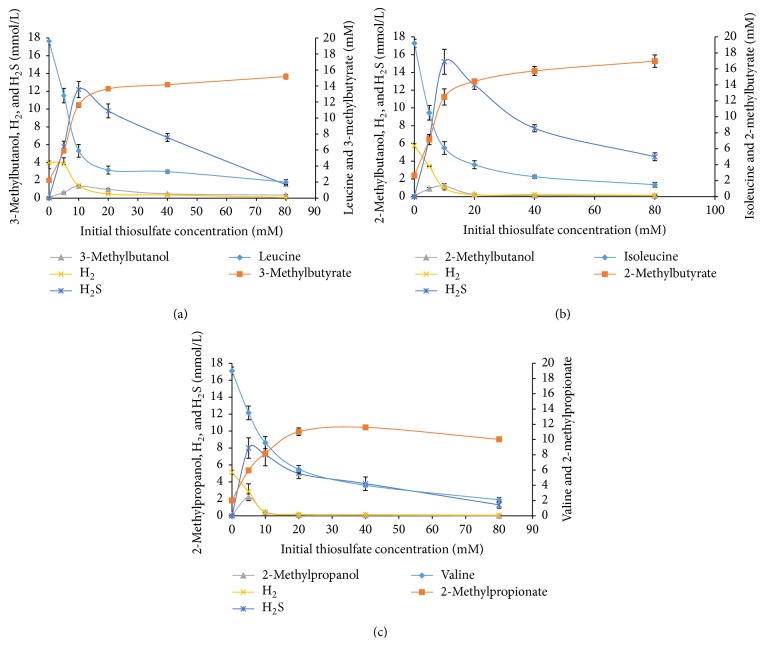
Amino acid degradation and end product formation at five different initial thiosulfate (5, 10, 20, 50, and 80 mM) concentrations by* Thermoanaerobacter brockii*. (a) Leucine degradation, (b) isoleucine degradation, and (c) valine degradation. Bars represent standard deviation from two replicates.

**Figure 3:**
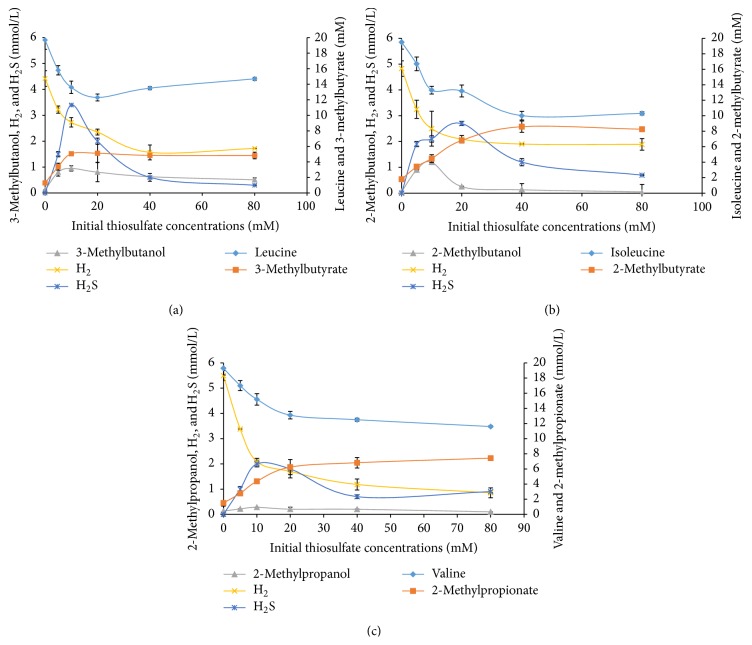
Amino acid degradation and end product formation at five different initial thiosulfate (5, 10, 20, 50, and 80 mM) concentrations by* Thermoanaerobacter ethanolicus*. (a) Leucine degradation, (b) isoleucine degradation, and (c) valine degradation. Bars represent standard deviation from two replicates.

The degradation of the BCAAs in the presence of thiosulfate by* T. brockii* and* T. ethanolicus* led to the production of both BCOHs and BCFAs, similar to strain AK90, though to a lesser extent (Figures 2 and 3). By increasing the initial concentrations of thiosulfate from 5 to 10 mM in* T. brockii* cultures on the three BCAAs, an increase of hydrogen sulfide was observed. However, by further increasing the thiosulfate concentrations to 20, 40, and 80 mM, the hydrogen sulfide concentrations did not increase in the experimental bottles; the hydrogen sulfide concentrations were, in fact, lower at higher thiosulfate loadings. The most reasonable explanation is either thiosulfate or H_2_S is converted to sulfur. Microscopic analysis revealed the presence of sulfur granules in the cultures and a strong yellow color formation was observed.

*T. ethanolicus* degraded only between 3.0 and 10.0 mM of the BCAA in the presence of thiosulfate. Additionally, hydrogen concentrations were higher at the end of incubation as compared with* T. brockii* and strain AK90. Thus,* T. ethanolicus* seems to be less effective in reducing thiosulfate, but hydrogen was found to be above 1 mmol L^−1^ even at very high thiosulfate concentrations. This is most likely the reason for incomplete BCAA degradation by this strain. The original characterization paper on* T. ethanolicus* indicated that the strain was incapable of utilizing yeast extract only, but its presence was crucial for sugar degradation [1]. Faudon and coworkers [25], however, showed that both* T. brockii* and* T. ethanolicus* were capable of peptide and amino acid degradation and that the presence of thiosulfate was indeed of importance resulting in more efficient degradation. However,* T. brockii* was much more efficient than* T. ethanolicus*, which is in line with our results.

Comparison of the three strains, AK90,* T. brockii*, and* T. ethanolicus*, shows that, under the same growth conditions (20 mM BCAA, 20 mM thiosulfate), strain AK90 produces most of the BCOH (from 1.8 to 4.4 mM) and* T. brockii* the least (between 0.1 and 1.0 mM) (Table 1; Figures 2 and 3). Strains AK90 and* T. brockii* produced between 9.7 and 19.2 mM of the BCFA from the BCAA, but* T. ethanolicus* only between 5.1 and 6.8 mM, which is reflected in lower BCAA degradation. Under methanogenic conditions, strains AK90 and* T. brockii* almost completely degraded the BCAA, which was also true for* T. ethanolicus* on both leucine and isoleucine, but not on valine (only 7.6 mM degraded) (Tables 1 and 2). This apparent ability to degrade valine by* T. ethanolicus* may be caused by differences in enzyme specificity.

The production of BCFAs and BCOHs from amino acids has been well established in yeasts such as* Saccharomyces cerevisiae* via the Ehrlich pathway [12, 32]. Recent investigations on the degradation of BCAAs to BCOHs by* Thermoanaerobacter brockii* and* Caldanaerobacter subterraneus* subsp.* yonseiensis* were recently reported [16]. This work led to a screening of various species within these two genera as well as within* Clostridium*,* Caldicellulosiruptor*,* Caloramator*, and* Thermoanaerobacterium* [17]. From these studies, only* Thermoanaerobacter* and* Caldanaerobacter* species showed the capacity to produce BCOHs from BCAAs. In both of these studies, it was clear that a factor of importance for BCOH formation was mainly the partial pressure of hydrogen [16, 17]. The present investigation shows that it is very likely that strain AK90 is also producing a mixture of aromatic fatty acids and aromatic alcohols during hydrogen-scavenging conditions.

The degradation of amino acids presents a renewable route to potentially important feedstock chemicals. The interest in BCAA degradation has often been directed towards the formation of flavor compounds (branched- and aromatic chain aldehydes, alcohols, and acids) in food and beverage products [12]. Additionally, (*S*)-2-methylbutanol is a potential biofuel [33] and some of the BCOHs may serve as building blocks [34]. Recently, some studies have focused on the production of branched-chain alcohols from protein-rich waste using genetically engineered* Escherichia coli* and* Bacillus subtilis* with the main focus being that BCOHs are promising biofuel candidates [35, 36].

## 5. Conclusion

*Thermoanaerobacter* strain AK90 degraded only serine when used as a single substrate but degraded nine amino acids under electron-scavenging conditions (thiosulfate and a coculture with a hydrogenotrophic methanogen). Branched-chain amino acids were degraded to their corresponding branched-chain fatty acids under methanogenic conditions and to a mixture of branched-chain fatty acids and alcohols under thiosulfate reducing conditions. This phenomenon was also exhibited by* Thermoanaerobacter brockii* (DSM 1457) and* Thermoanaerobacter ethanolicus* (DSM 2246), though to a lesser extent. The formation of these end products seems to be highly dependent upon partial pressure of hydrogen.

## Figures and Tables

**Table 1:** End product formation from amino acids by *Thermoanaerobacter *strain AK90. Initial amino acid concentration was 20 mM in all cases. Experiments were done with and without electron-scavenging systems, either with 40 mM of thiosulfate (S_2_O_3_^2−^) or in a coculture with a hydrogenotrophic methanogen (M39). Data represent the average of two replicate experiments ± standard deviation. Experiments without added carbon source only contained 2 g/L of yeast extract (YE). Branched-chain fatty acids (BCFAs) are 3-methylbutyrate from leucine, 2-methylbutyrate from isoleucine, and 2-methylpropionate from valine. Branched-chain alcohols (BCOHs) are 3-methylbutanol from leucine, 2-methylbutanol from isoleucine, and 2-methylpropanol from valine.

Substrate/conditions	Amino acids (end of fermentation)	End products (mmol/L)							Carbon balance (%)
		Ethanol	Acetate	BCFA	BCOH	H_2_	H_2_S	CH_4_	
YE	ND^1^	2.1 ± 0.1	5.2 ± 0.1	0.4 ± 0.1^2^	<0.1^3^	7.1 ± 0.2	ND	ND	ND
YE + S_2_O_3_	ND	2.5 ± 0.2	8.9 ± 0.3	0.9 ± 0.1^2^	0.1 ± 0.0^3^	<0.1	0.3 ± 0.1	ND	ND
YE + M39	ND	1.0 ± 0.1	8.7 ± 0.2	0.9 ± 0.2^2^	<0.1^3^	<0.1	ND	2.2 ± 0.1	ND

Ala	19.2 ± 1.5	1.2 ± 0.1	3.4 ± 0.3	<1.0	<0.1	10.9 ± 0.2	ND	ND	ND
Ala + S_2_O_3_	0.0 ± 0.0	5.5 ± 0.6	25.4 ± 0.8	<1.0	<0.1	1.3 ± 0.2	11.5 ± 0.8	ND	97.5^4^
Ala + M39	6.1 ± 1.6	1.4 ± 0.1	21.4 ± 2.8	<1.0	<0.1	<0.1	ND	8.0 ± 0.1	94.2^4^

Cys	19.3 ± 0.0	1.2 ± 0.1	3.4 ± 0.1	<1.0	<0.1	6.3 ± 0.5	ND	ND	ND
Cys + S_2_O_3_	0.2 ± 0.1	3.0 ± 1.1	24.2 ± 1.6	<1.0	<0.1	1.6 ± 0.1	12.9 ± 0.4	ND	79.8^4^
Cys + M39	0.0 ± 0.0	1.5 ± 0.1	25.6 ± 1.7	<1.0	<0.1	<0.1	ND	8.6 ± 0.4	87.0^4^

Ile	16.2 ± 0.4	1.4 ± 0.3	3.0 ± 0.5	3.8 ± 0.2	<0.1	11.1 ± 0.9	ND	ND	ND
Ile + S_2_O_3_	0.0 ± 0.0	2.9 ± 1.2	8.1 ± 1.1	15.9 ± 0.7	4.4 ± 0.3	0.2 ± 0.0	12.5 ± 0.6	ND	100.0
Ile + M39	0.0 ± 0.0	1.4 ± 0.3	8.4 ± 1.4	15.6 ± 1.6	0.2 ± 0.0	0.0 ± 0.0	ND	11.7 ± 1.3	77.5

Leu	18.5 ± 0.4	4.9 ± 0.5	4.2 ± 0.3	2.0 ± 0.1	<0.1	8.9 ± 0.3	ND	ND	ND
Leu + S_2_O_3_	0.0 ± 0.0	2.9 ± 1.0	8.1 ± 0.4	9.7 ± 2.1	3.5 ± 0.3	0.6 ± 0.3	13.5 ± 0.7	ND	64.5
Leu + M39	4.0 ± 0.4	1.3 ± 0.2	4.3 ± 0.7	11.7 ± 0.4	0.4 ± 0.1	<0.1	ND	9.9 ± 0.3	73.1

Lys	20.0 ± 0.0	1.1 ± 0.1	2.6 ± 0.1	<1.0	<0.1	5.8 ± 0.1	ND	ND	ND
Lys + S_2_O_3_	15.9 ± 2.2	3.7 ± 0.4	9.9 ± 0.5	<1.0	<0.1	0.1 ± 0.0	2.2 ± 0.4	ND	ND^5^
Lys + M39	8.5 ± 1.2	1.6 ± 0.2	19.5 ± 6.2	<1.0	<0.1	<0.1	ND	5.5 ± 0.3	ND^5^

Met	19.5 ± 0.5	3.0 ± 0.9	4.0 ± 0.2	<1.0	<0.1	8.2 ± 0.4	ND	ND	ND
Met + S_2_O_3_	3.6 ± 0.5	3.0 ± 0.3	6.1 ± 0.3	<1.0	<0.1	0.7 ± 0.1	12.4 ± 0.9	ND	ND
Met + M39	0.4 ± 0.1	1.8 ± 0.2	9.7 ± 0.7	<1.0	<0.1	<0.1	ND	12.2 ± 0.6	ND

Phe	20.4 ± 0.6	1.1 ± 0.1	2.6 ± 0.1	<1.0	<0.1	8.0 ± 0.1	ND	ND	ND
Phe + S_2_O_3_	8.3 ± 1.0	2.8 ± 0.3	6.3 ± 0.4	<1.0	<0.1	1.4 ± 0.1	7.4 ± 0.6	ND	ND
Phe + M39	0.1 ± 0.0	1.5 ± 0.2	10.4 ± 1.1	<1.0	<0.1	<0.1	ND	11.7 ± 0.4	ND

Ser	4.0 ± 0.5	5.7 ± 0.5	15.7 ± 0.6	<1.0	<0.1	9.7 ± 0.5	ND	ND	87.5^4^
Ser + S_2_O_3_	0.0 ± 0.0	3.3 ± 1.1	26.8 ± 1.4	<1.0	<0.1	0.1 ± 0.1	12.5 ± 1.2	ND	93.5^4^
Ser + M39	0.0 ± 0.0	1.5 ± 0.3	27.5 ± 2.0	<1.0	<0.1	<0.1	ND	8.8 ± 0.2	96.5^4^

Thr	19.0 ± 1.3	1.1 ± 0.1	2.6 ± 0.1	<1.0	<0.1	8.0 ± 0.8	ND	ND	ND
Thr + S_2_O_3_	14.5 ± 2.5	3.0 ± 0.4	25.4 ± 2.1	<1.0	<0.1	0.1 ± 0.0	11.5 ± 1.0	ND	ND^6^
Thr + M39	1.5 ± 0.2	0.8 ± 0.1	31.2 ± 1.8	<1.0	<0.1	<0.1	ND	6.9 ± 0.5	ND^6^

Tyr	19.3 ± 0.5	1.1 ± 0.1	2.6 ± 0.1	<1.0	<0.1	6.3 ± 0.5	ND	ND	ND
Tyr + S_2_O_3_	6.8 ± 1.3	3.4 ± 0.1	7.7 ± 0.3	<1.0	<0.1	0.9 ± 0.2	9.5 ± 0.3	ND	ND
Tyr + M39	1.2 ± 0.2	1.4 ± 0.1	10.0 ± 1.1	<1.0	<0.1	<0.1	ND	7.1 ± 2.0	ND

Val	17.4 ± 1.0	1.1 ± 0.1	2.6 ± 0.1	3.3 ± 0.1	<0.1	9.5 ± 0.1	ND	ND	ND
Val + S_2_O_3_	0.0 ± 0.0	3.2 ± 1.1	7.6 ± 0.6	19.2 ± 1.0	1.8 ± 0.5	0.6 ± 0.2	12.4 ± 0.5	ND	103.5
Val + M39	3.3 ± 1.7	1.4 ± 0.0	6.4 ± 1.6	12.3 ± 2.4	<0.1	<0.1	ND	8.9 ± 2.0	71.8

^1^ND: not determined.

^2^Total of 3-methylbutyrate, 2-methylbutyrate, and 2-methylpropionate.

^3^Total of 3-methylbutanol, 2-methylbutanol, and 2-methylpropanol.

^4^Assuming that CO_2_ is produced in equimolar ratio with the production of acetate and ethanol.

^5^Butyrate was produced (2.2 mM) but not shown in the table for simplicity reasons.

^6^Degradation pathway unknown and thus not calculated (see results and discussion).

**Table 2:** Amino acid degradation and end product formation from branched-chain amino acids by *Thermoanaerobacter brockii *and* Thermoanaerobacter ethanolicus *in a coculture with *Methanothermobacter* strain M39. Initial amino acid concentration was 20 mM in all cases. Data represent the average of two replicate experiments ± standard deviation. The branched-chain fatty acids from leucine, isoleucine, and valine were 3-methylbutyrate, 2-methylbutyrate, and 2-methylpropionate, respectively. ^#^Data from [16].

Strain and substrates	Amino acid (end of cultivation)	Branched-chain fatty acid	Methane
	Concentration (mmol/L)		
*T. brockii*: leucine^#^	1.5 ± 0.2	15.9 ± 2.3	9.3 ± 0.8
*T. brockii*: isoleucine^#^	1.7 ± 0.3	15.4 ± 1.3	8.3 ± 0.8
*T. brockii*: valine^#^	2.4 ± 0.2	14.8 ± 0.9	7.6 ± 1.0

*T. ethanolicus*: leucine	3.5 ± 0.3	15.0 ± 1.3	7.4 ± 1.1
*T. ethanolicus*: isoleucine	2.7 ± 0.4	14.3 ± 0.8	7.8 ± 0.9
*T. ethanolicus*: valine	12.4 ± 0.8	7.6 ± 0.6	3.4 ± 1.3
